# KTN (RCK) Domains Regulate K^+^ Channels and Transporters by Controlling the Dimer-Hinge Conformation

**DOI:** 10.1016/j.str.2009.03.018

**Published:** 2009-06-10

**Authors:** Tarmo P. Roosild, Samantha Castronovo, Samantha Miller, Chan Li, Tim Rasmussen, Wendy Bartlett, Banuri Gunasekera, Senyon Choe, Ian R. Booth

**Affiliations:** 1 Drug Development Department, Nevada Cancer Institute, Las Vegas, NV 89135, USA; 2 School of Medical Sciences, Institute of Medical Sciences, University of Aberdeen, Aberdeen AB25 2ZD, Scotland, UK; 3 Structural Biology Laboratory, Salk Institute, La Jolla, San Diego, CA 92037, USA

**Keywords:** PROTEINS, SIGNALING

## Abstract

KTN (RCK) domains are nucleotide-binding folds that form the cytoplasmic regulatory complexes of various K^+^ channels and transporters. The mechanisms these proteins use to control their transmembrane pore-forming counterparts remains unclear despite numerous electrophysiological and structural studies. KTN (RCK) domains consistently crystallize as dimers within the asymmetric unit, forming a pronounced hinge between two Rossmann folds. We have previously proposed that modification of the hinge angle plays an important role in activating the associated membrane-integrated components of the channel or transporter. Here we report the structure of the C-terminal, KTN-bearing domain of the *E. coli* KefC K^+^ efflux system in association with the ancillary subunit, KefF, which is known to stabilize the conductive state. The structure of the complex and functional analysis of KefC variants reveal that control of the conformational flexibility inherent in the KTN dimer hinge is modulated by KefF and essential for regulation of KefC ion flux.

## Introduction

Potassium (K^+^) channels and transporters play central roles in the maintenance of cellular homeostasis, including osmolarity and pH, and in higher organisms have been adapted for swift, yet precise, nerve impulse propagation. The regulation of these channels, a process referred to as gating, is often performed by cytoplasmic domains that respond to cellular cues to alter ion flux through the pore. In addition to channels, cellular K^+^ levels are maintained by the closely regulated balancing of import and export by a variety of ion transporters. In prokaryotes, KTN domains (K^+^ transport, nucleotide binding; Roosild et al., 2002) are ubiquitous among the major K^+^ selective transport systems: uptake (Trk and Ktr; Epstein, 1986; Schlosser et al., 1993; Nakamura et al., 1998; Kröning et al., 2007), channels (Kch; Derst and Karschin, 1998]), and efflux (Kef; Munro et al., 1991). These domains, closely related to RCK domains (Jiang et al., 2001), are found either within a cytoplasmic subunit (TrkA or KtrA; Bossemeyer et al., 1989; Nakamura et al., 1998) or covalently linked to the C terminus of the pore-forming transmembrane domain (Kch or Kef; Munro et al., 1991), analogous to this domain's positioning in eukaryotic large-conductance, calcium-activated (BK) channels (Jiang et al., 2001). The universal distribution of this domain throughout a diverse range of K^+^ transport systems and their invariant physical proximity to the base of the innermost pore-forming helices, supports that they play a central role in the regulation and coordination of K^+^ flux across cellular membranes.

Several KTN (RCK) domain structures have now been determined (Jiang et al., 2001, 2002; Roosild et al., 2002; Dong et al., 2005; Ye et al., 2006; Albright et al., 2006; Kuo et al., 2007). Without exception, in each case the solved structure has contained KTN (RCK) dimers, with the sixth α helix of a Rossmann fold swapped between adjoining domains. However, the functional biological unit has remained unclear. Analysis of the solution stoichiometry of KTN domains from KtrA in the presence of NAD(H) ligand has suggested a tetrameric structure in vivo (Roosild et al., 2002). In contrast, serendipitous tail-to-tail crystallization of an MthK channel variant (M107I; Jiang et al., 2002) bearing a C-terminal RCK domain led to an octameric ring hypothesis. It was proposed that an octameric structure in the native channel would arise by translation of the RCK domain from an internal methionine within MthK's open reading frame, creating separate and soluble proteins that interlaced noncovalently with the RCK domains of the pore-forming, full-length channel proteins. A more recent study (Albright et al., 2006) resolved the structure of the KtrA subunit from *B. subtilis* and concluded that this protein also forms octameric rings that undergo conformational transitions through three distinct crystallographic symmetries. Surprisingly, they also suggested that two transmembrane pores associate with the ring in this system, in stark contrast to the original one ring/one pore model developed with the MthK channel. However, some functional studies have revealed additional complexity to the KTN (RCK) regulatory mechanism: elimination of the putative internal translation start site did not alter channel expression or function of *E. coli* Kch (Kuo et al., 2003), and electrophysiological studies of MthK channels have shown that stoichiometric variation is integral to channel regulation (Kuo et al., 2007, 2008).

**Figure 1:**
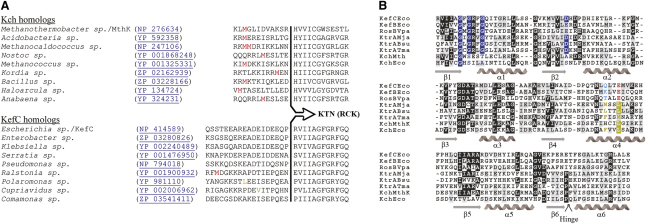
Sequence Analysis of KTN (RCK) Domains (A) Shown is the sequence of the linker region preceding the KTN (RCK) domain in eight BLAST-identified homologs of MthK and KefC from distinct genera. The Kch proteins invariably contain methionine residues (red) that might potentially initiate translation internal to the larger open reading frame. In contrast, Kef linkers do not have this feature, suggesting an inability to create such supplementary, cytoplasmic copies of the KTN (RCK) domain in isolation. A CTG codon that can in some cases initiate translation is colored orange. The sole valine (green) is not coded by a recognized initiation codon. (B) Structure-based sequence alignment of KTN (RCK) domains reveals that whereas Kef systems strictly conserve the Rossmann motif residues (blue), implying retention of the capacity to bind nucleotides, they do not have the surface exposed hydrophobic residues (yellow) on α helix 4 that mediate dimer-dimer oligomerization in other KTN (RCK)-bearing proteins.

We have approached this question by studying the KTN domain from a third, unique system, namely Kef. Notably, Kef efflux systems, though having C-terminally covalently linked KTN domains similar to canonical K^+^ channels, do not possess appropriately positioned internal translation initiation codons to produce supplemental, soluble KTN-bearing proteins for ring assembly (Figure 1A). Additionally, Kef KTN domains lack the surface-exposed hydrophobic patches that have been previously shown in other proteins to mediate dimer-dimer assembly (Figure 1B). These characteristics suggest that new insights into the mechanisms by which KTN (RCK) domains control ion flux might be revealed by structural analysis of KTN-containing assemblies from Kef systems.

Kef transporters are those members of the monovalent cation:proton antiporter-2 superfamily of proteins characterized by K^+^ selectivity and KTN regulation. Their transmembrane domains are distantly related to other cation:proton antiporters, the best studied of which is *E. coli* NhaA, which has recently been confirmed to have a dimeric native architecture (Hilger et al., 2007; Appel et al., 2009). Many of these proteins, including *E. coli* KefC and KefB, are glutathione (GSH)-gated K^+^ efflux systems that are usually maintained in a closed state. Exposure of the cell to electrophiles leads to the formation of GSH adducts (GSX) that are activators of the efflux system (Elmore et al., 1990; Ferguson et al., 1995). Activation of the efflux system leads to cytoplasmic acidification mediated by H^+^ charge compensation for K^+^ expulsion that in turn protects the organism's DNA from electrophilic attack and damage (Ferguson et al., 2000). Overall, this improves the organism's ability to resist and detoxify harmful metabolites such as methylglyoxal. In contrast to other KTN (RCK)-bearing channels and transporters, the KefC system has an additional level of complexity involving an ancillary subunit, KefF (Miller et al., 2000), with sequence homology to flavin-binding quinone oxidoreductases (QR1 and QR2; Li et al., 1995; Foster et al., 1999; Faig et al., 2000) and microbial modulators of drug activity (MdaB; Adams and Jia, 2006). KefF is essential for full activation of the KefC system and therefore appears to be an integral part of the system's gating machinery. Importantly, however, the residual activity of KefC in the absence of KefF retains sensitivity to GSH and GSX (Miller et al., 2000), suggesting two separate regulatory mechanisms, possibly coupled through the KTN domain. These features of the KefC antiporter make it a tractable model for studying the KTN (RCK) regulatory mechanism as activating ligands (i.e., GSX, formed by conjugation of GSH with electrophilic compounds such as N-ethylmaleimide [NEM] or methylglyoxal; Elmore et al., 1990), inhibitory ligands (GSH and NADH; Fujisawa et al., 2007), and an activating protein subunit (KefF) are very well characterized through in vivo studies in the native organism, and can be used in biophysical studies to stabilize specific conformations of the regulatory complex. Further, genetic and functional assays can be performed in vivo to complement structural analysis and support structure-guided hypotheses through biological phenotype characterization.

In this report, we present the structure of the KTN-bearing C-terminal domain of *E. coli* KefC (KefC-CTD) in complex with KefF, resolved to 2.4 Å resolution. The structural and functional analysis of this assembly support the proposal that KTN (RCK) domains operate by mediating directed control of the conformation of the dimer-hinge angle formed between two KTN domains. The structure also reveals the binding site for GSH ligands and suggests a mechanism for channel regulation by these compounds. These results demonstrate that octameric organization of KTN (RCK) domains is not functionally relevant to regulating KefC in vivo.

## Results

### KefC Regulatory Domains Are Dimeric

**Figure 2:**
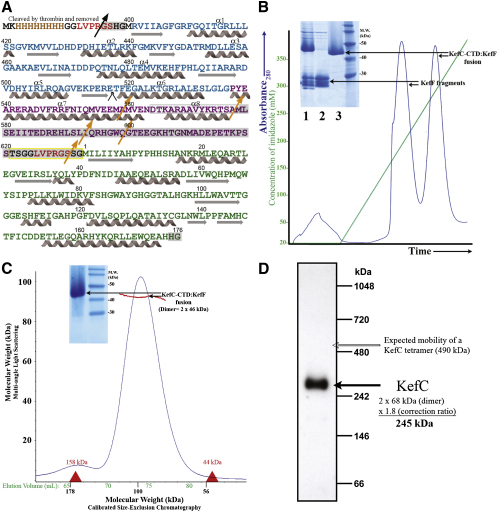
Analysis of KefC-CTD:KefF Fusion Protein (A) Shown is the primary sequence of the fusion construct of KefC-CTD containing a KTN domain (blue) with KefF (green), highlighting proteolytically susceptible points (orange arrows) in the very C terminus of the KefC transporter (purple). Locations of a polyhistidine tag for purification (brown) and thrombin cleavage motifs (red) are also illustrated. Helices in KefC-CTD discussed in the report are labeled. Residues disordered in the crystal structure are shaded gray (those residues that are disordered in only one of the two monomers are hatched). The residues comprising the linker from KefC to KefF are highlighted in yellow. (B) SDS-PAGE shows high yields of fusion protein after Ni-NTA affinity purification with some contamination by truncated forms of the protein (lane 1). The contaminants are KefF fragments (lane 2) that can be removed utilizing a shallow imidazole gradient (green line) for elution from Ni-NTA resin, yielding pure, full-length regulatory complex (lane3). (C) Calibrated size-exclusion chromatography (molecular weight standards indicated in red) of the fusion protein reveals a monodisperse, dimeric species with no evidence of higher-order assembly or aggregation. SDS-PAGE analysis of the final sample (inset) reveals only homogenous fusion protein. Multiangle light scattering analysis (red) with refractive index measurement, in line with size-exclusion chromatography, confirms the KefC-CTD:KefF fusion protein is dimeric in solution. (D) Blue native-PAGE of whole KefC channels (without KefF) directly solubilized from the bacterial membrane using the detergent dodecylmaltoside (DDM) confirms retention of a dimeric assembly in vivo. Shown is a western blot conducted with anti-His6 antibodies for detection of recombinant KefC analyzed using a factor of 1.8 for the true mass:mobility-based mass ratio in accordance with Heuberger et al. (2002).

Initial attempts to express the KefC-CTD domain alone or with KefF failed to produce homogenous, soluble protein. In order to stabilize the KefC-CTD/KefF complex for structural analysis, fusion of the two domains via a flexible 12-residue synthetic linker was pursued. The fusion construct, KefC-CTD:KefF (Figure 2A), yielded high-level expression of a bright yellow protein with significant susceptibility to proteolytic degradation (Figure 2B, inset). Breakage points were identified utilizing a combination of mass spectrometry and N-terminal protein sequencing, revealing their location at several points within the poorly conserved, very C-terminal peripheral domain of KefC. Unexpectedly, all of the truncated bands belong to the half of the fusion containing the KefF domain and lack a polyhistidine affinity tag. Presumably, the reciprocal KefC-CTD fragment is lost by aggregation, precipitation, or further degradation, and therefore is not present in these samples. Based on this observation, a shallow gradient of imidazole was used during elution of the protein from Ni-NTA resin to separate all of the fragments from the whole fusion protein (Figure 2B). Pure complex was analyzed by gel filtration and multiangle light scattering analysis, and was found to be monodisperse and homodimeric without regard to the presence or absence of antagonistic or agonistic ligands (Figure 2C). Consistent with this observation, full-length recombinant KefC analyzed by blue native gel electrophoresis gave a mass of around 270 kDa (Figure 2D), which is approximately the predicted mass of a dimer of KefC in association with Coomassie blue dye (Heuberger et al., 2002).

### KefF Stabilizes an Open Conformation of the KTN Hinge

The crystal structure of the KefC-CTD/KefF complex (Table 1) was determined at 2.4 Å resolution by molecular replacement, using known structures of quinone reductases with strong sequence similarity to KefF and solved KTN-bearing structures as models for KefC-CTD. Crystals contain one dimer of the fusion complex within the asymmetric unit. Although all of the residues of KefF could be constructed from the resulting electron density maps, much of the C-terminal peripheral domain of KefC-CTD, as well as all of the artificial linker, are disordered. There is additionally some asymmetry between the two monomers, as α8 of KefC-CTD could only be clearly visualized in one monomer, and must possess an alternate conformation in the partnering chain due to steric interference by a symmetry-related protein molecule within the crystal lattice.

**Figure 3:**
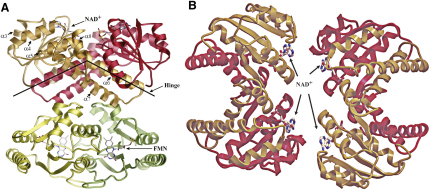
Structure of KefC-CTD in Complex with KefF (A) The KefC-CTD KTN domains form a canonical hinged dimer, with a very open crossing angle (red and orange). KefF interfaces under the hinge through contacts with the loops at the ends of each arm, directly influencing hinge conformation. Helices in KefC-CTD discussed in the report and cofactors are labeled. (B) In trigonal crystals of KefC-CTD:KefF, two dimers associate to form a dimer-of-dimers tetrameric complex, resembling models hypothesized for KtrA tetramers (KefF not shown). Although such assemblies might not form in vivo for KefC antiporters, the observed quaternary structure provides additional insights as to how KTN (RCK) domains might interact in systems like canonical K^+^ channels, where tetrameric transmembrane domains force proximate localization of 4 KTN (RCK) folds in the cytoplasm.

**Figure 4:**
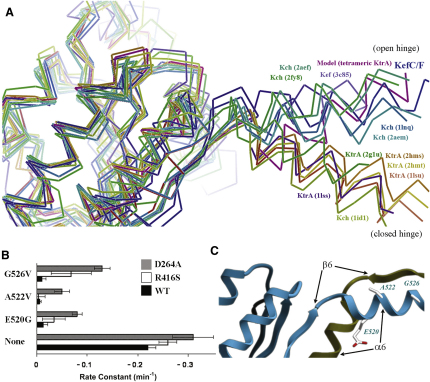
Hinge Conformations of KTN (RCK) Structures (A) Alignment of all known, unique KTN (RCK) structures (one monomer of the canonical dimer shown) reveals a two-state system, with the hinge either open to ∼120° or closed to ∼90°. These two conformations likely correspond to open and closed states for the associated channel or transporter. (B) KefC activity can be suppressed by a series of mutations within the KTN domain. These mutations are more effective at suppressing the spontaneous activity of KefC variants mutated within the KTN domain (R416S) than within the pore-forming transmembrane domain (D264A). (C) Ribbon representation of KefC from the same approximate view point as in (A) reveals that these second site suppressors (E520G, A522V, G526V) of mutations that cause spontaneous KefC activity (such as R416S; not shown) cluster near the hinge formed by the domain swapping of α-helix 6.

The structure reveals that KefF binds to the ends of the hinge-forming arms of KefC's KTN domain, fixing this hinge at approximately 120° (Figure 3A). These results suggest that KefF functions through directly influencing this angle, potentially stabilizing a conductive state of the channel, thereby explaining the dependence of rapid KefC ion flux on KefF binding (Miller et al., 2000). A second low-resolution crystal form, solved by rigid-body molecular replacement and refinement, verifies the same global conformation, but has two KTN dimers facing each other to form a “dimer of dimers” assembly that is highly similar to the tetrameric form of KtrA proposed previously (Figure 3B; Roosild et al., 2002). Although this assembly mode might not be biologically relevant for the KefC system, it reflects a possible association mechanism for tetramer formation in other KTN (RCK) systems. An analysis of all known KTN (RCK) structures, including this domain from *T. maritima* KtrA and a *V. parahaemolyticus* Kef homolog (both unpublished depositions from structural genomics consortia; Joint Center for Structural Genomics, 2006; Wu et al., 2008) reveals a discreet two-state system with the KTN (RCK) hinge at either a 90° or 120° angle (Figure 4A), suggesting that this is a critical element of the KTN (RCK) gating mechanism.

To verify this hypothesis, we sought supporting evidence from a genetic analysis of suppressors of a spontaneously active KefC mutation. We have previously identified several missense mutations in the KefC-CTD that modify the regulation of the system leading to a high rate of spontaneous K^+^ loss (Miller et al., 1997; Ness and Booth, 1999). Suppressors were sought to two of these KTN domain mutations (V427A and R416S) utilizing restoration of growth on low K^+^ agar as a selection to find mutations that arise to block KefC activity. Such suppressors were subsequently screened for retention of KefC activity to filter out those mutations that simply block expression of the full-length protein, which results in the same phenotype. Each suppressor was sequenced and the mutation re-created in wild-type KefC, in an R416S derivative of KefC, and in a KefC protein modified in the regulatory HALESDIEP sequence that lies in the intratransmembrane helix loop between α7 and α8 of the transporter domain and contributes to the regulation of the conductance state of KefC. All identified suppressor mutations lowered the activity of KefC (activated by formation of ESG; Elmore et al., 1990) independent of whether the mutation was in a wild-type or modified KefC framework (Figure 4B). Consistently, these suppressors fell into two primary classes: those that disrupt the expression or folding of KefF (Q62L and L95R; data not shown), and point mutations in KefC under the KTN dimer hinge (E520G, E520K, A522V, and G526V; Figure 4C). In fact, the only hinge-region mutation isolated and analyzed that retained any activity was a double mutant, E520I/K524Y, which possesses ∼10% of parental activity (data not shown); this double mutant replicates the sequence at this point in KtrA of *M. jannaschii* (Roosild et al., 2002). Even a charge-switching double mutant that reverses the polarity of an intrahelical salt bridge (E520K/K524E) loses all activity (data not shown). With the solution of the structure of the complex between these two proteins, it is now clear as to how these suppressors might be mechanistically linked as both classes affect the KTN angular conformation: KefF fold-destabilizing mutations prevent this ancillary subunit from bracing the open-hinge K^+^-conducting conformation of the KTN domain, whereas hinge-localized mutations affect the energetics of hinge motion directly.

### KefC/KefF Interface and KefF Active Site Structure

**Figure 5:**
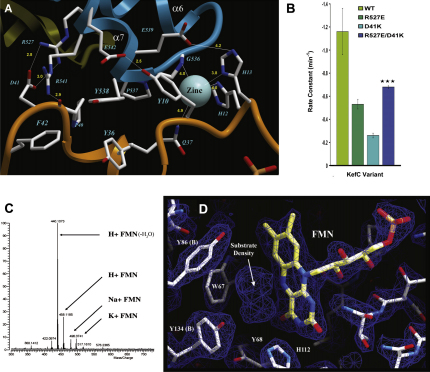
Structure of the KefF/KefC Interface and KefF Active Site Features (A) The interface between KefF (orange) and KefC (blue) is formed primarily by residues from the KefC-CTD between α helices 6 and 7, representing the ends of the arms in the hinged structure of this protein. The molecular interactions include a coordinated zinc ion and a salt-bridge through R527, a residue previously identified to be critical for channel activation. (B) Although mutations R527E in KefC and D41K in KefF have reduced activity, similar to that observed for KefF destabilizing mutations (Q62L, L95R; not shown), the simultaneous mutation of both proteins (reforming the salt-bridge with opposite polarity) partially restores activity to the KefC system, suggesting that the interface between the two proteins as seen in the structure accurately reflects the physiological association between them. Data were subjected to a Student's t test with single tails and two sample equal variance, generating a p value of 0.001. Note that the higher rate constants for KefC/KefF activity, compared with Figures 4 or 6, arise from the use of an expression construct that here bears both *kef*F and *kef*C. (C) Mass spectroscopy analysis of eluted flavin cofactor from urea denatured KefF reveals its identity as flavin mononucleotide (FMN). (D) The active site of KefF shows conservation of quinone reductase structure and catalytic residues (in comparison to human QR1, W67 is strictly conserved and Y86, Y134 and Y68 are all phenylalanine; all four residues are nearly identically positioned in the two proteins). Bound FMN is also clearly seen in the electron density maps (2F_o_-F_c_ shown contoured at 1.5σ), and additional density is visible in the location where substrates would be expected to bind.

Unexpectedly, the structure reveals that KefF has a novel zinc binding site not found in the other quinone reductase enzymes with which it shares homology. The metal cation is bound primarily by three KefF residues: H12, H13, and Q37 (Figure 5A). Further, this zinc forms part of the extensive interface between the two subunits of the Kef system, being also coordinated by a backbone amide group belonging to G536 of KefC. Other characteristics of this interface are very typical of conventional protein-protein contacts with a hydrophobic core formed by Y538 of KefC intercalating into a pocket lined by Y10, Y36, and F42 of KefF, and numerous additional polar contacts surrounding this nucleus. Additionally, KefC R527, a residue that had been previously shown to be critical for KefC activity, forms a salt bridge with D41 of the KefF subunit. Analysis of KefF D41K revealed a disabled transport system phenotypically similar to KefF knockout. Further, the reduced activity of the D41K mutant could be partially suppressed by the complementing mutation of KefC R527E (Figure 5B). The extensive nature, congruent chemistry, and coherent physiology of the KefC/KefF interface suggest that this fusion construct structure accurately reproduces the in vivo association of these two protein partners.

The structure of the KefF substrate binding pocket is remarkably well conserved with other quinone reductases of known structure, suggesting a possible retention of activity. The cofactor for this protein was determined to be FMN by mass spectroscopy (Figure 5C), a conclusion that is consistent with the observed electron density in the structure (Figure 5D). The structure also reveals extensive additional density within the pocket, suggesting a bound compound (Figure 5D). Given the composition of the crystallization solution, this might be the nicotinamide ring from NAD^+^ (either degraded or with the remainder of the ligand disordered) or some other molecule retained throughout isolation and purification of the protein.

### Novel Helix-Turn-Helix Arm Forms a Ligand Response Mechanism

**Figure 6:**
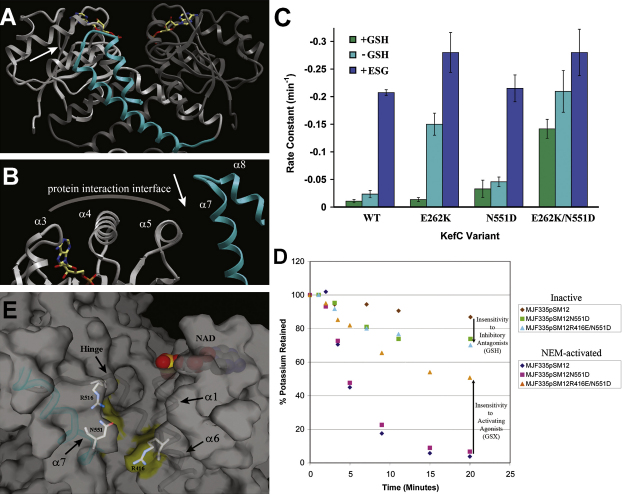
Mechanism of Glutathione Modulation of Channel Activity (A) KefC-CTD possesses a long helix-turn-helix structure (α7-α8; blue) that is not present in other KTN (RCK) proteins. (B) This arm, now viewed as from the arrow in A, protrudes into the protein interaction interface of the KTN domain that has been shown to be critical for channel regulation in related systems. This positioning suggests a role for this novel structural feature in mediating protein-protein contacts between the regulatory KTN fold and some other portion of the protein, including potentially the physical gate of the channel. (C) Analysis of E262K, a mutation occurring within the HALESDIEP intratransmembrane KefC gating loop that sensitizes the transporter to both agonists and antagonists, helps identify residues involved in ligand binding. Shown are the rate constants for KefC K^+^ efflux with and without the presence of the N551D mutation that weakens transporter inhibition by GSH. Although GSH is able to inhibit K^+^ leakage in the single-site E262K variant, proteins with N551D leak substantially without regard to GSH concentration. (D) Functional analysis of KefC mutants reveals progressive loss of sensitivity to inhibitory glutathione (GSH) and activating GSH adducts (GSX) with compounding changes in the residues lining the putative ligand binding pocket (N551D, R416E). (E) Helix 7 (blue), now viewed as from the arrow in B, creates a novel, deep pocket beside the KTN domain that is lined with all of the residues that have been identified through functional assays to alter the activity of GSH and its adducts with regard to transporter activation (R416, R516, N551).

All known structures of KTN (RCK) domains consist of hinged dimers (within the asymmetric subunit) that often adjoin other crystallographic dimers through an extensive, flat, and significantly hydrophobic protein-protein association surface critical for channel activity (Kim et al., 2006). This region, formed by the third through fifth α helices of the domain, has alternately been called in literature the “flexible” or “rotating” interface (hereafter it will be referred to as the protein interaction interface). Surprisingly, our structure displays no significant protein-protein contacts around this critical area. Instead, the very long seventh α helix and fairly mobile eighth α helix, comprised from the C-terminal residues of KefC beyond the KTN fold, protrude adjacent to this surface, disrupting the potential to form protein-protein interactions similar to those seen in the previous structures (Figures 6A and 6B). These residues are structured completely different from those of MthK and cannot be compared with the C-terminal structure of KtrA, because this part of those proteins has been removed before crystallization and remains structurally uncharacterized (Roosild et al., 2002; Albright et al., 2006).

**Figure 7:**
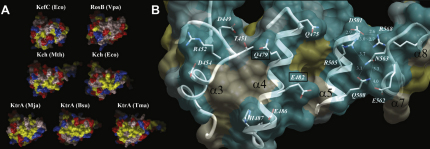
Surface Properties of KTN (RCK) Domains (A) Comparison of the structures of KTN (RCK) domains reveals that, whereas Kch and KtrA proteins possess a hydrophobic surface-exposed patch at the protein interaction interface (yellow: AVILMFP), Kef proteins have evolved to have a more polar composition in this region (red: ED; blue: RK; remainder: gray), suggesting that they do not form oligomers through homomultimeric protein-protein contacts in a manner similar to the other two permeases. (B) An illustration of the surface-exposed residues of the protein interaction interface of KefC is shown. In Kef channels, the central residues of this region are conserved as hydrophilic amino acids (such as Q479 and E482 for KefC; boxed), suggesting adaptation to a constitutively dimeric regulatory unit.

Mutations that cause KefC to have reduced sensitivity to inhibition by GSH (R416X and R516X) were identified as mutations causing a rapid spontaneous loss of K^+^ via KefC (Miller et al., 1997) or were found to affect K^+^ retention of an E262K mutation in the HALESDIEP sequence (N551D). Although the double mutation, N551D/E262K, exhibits a rapid K^+^ leak, the E262K mutation in isolation does not leak K^+^ unless the cells are depleted of GSH (Figure 6C). The N551D mutation alone exhibits a slow K^+^ leak that is not enhanced by removal of GSH, consistent with a lack of inhibition by this ligand in this mutant protein (Figure 6C). All of these residues map to a discreet crevice (∼12 Å in length) created by residues contributed from both subunits of the dimer (R416:A, R516:B, and N551:B). Notably, these residues also reside in close proximity to the hinge with R516 at the very C-terminal end of β6. Further, an N551D/R416E double mutant not only exhibits the expected K^+^ leak without regard to the presence of GSH, but is also insensitive to activation by NEM (Figure 6D). The spatial clustering of these three residues that are critical for ligand response in KefC reveals that the central part of the long α7 helix creates a GSH binding pocket with the KTN domain (Figure 6E). In aggregate, these observations suggest that this new helix-turn-helix structure is critical to the transporter's ability to sense and respond to GSH-derived ligands. It is also of note, that the protein interaction interface of KefC's KTN domain is substantially more polar in comparison to previously studied KTN (RCK) structures (Figure 7). This adaptation might facilitate its interaction with the physical gate of the channel, which is believed to be formed by a highly-charged, cytoplasmic-facing, intra-transmembrane helix motif (in *E. coli* KefC it is “HALESDIEP”; Miller et al., 1997; Ness and Booth, 1999).

## Discussion

Despite numerous structural studies, the mechanism by which KTN (RCK) domains regulate associated K^+^ transporters and channels remains uncertain. In this report we address this question by studying a KTN domain from a representative of a structurally uncharacterized third system. We had originally hypothesized that regulation of hinge conformation was the primary mechanism for KTN (RCK) control of K^+^ flux through its associated channel or transporter (Roosild et al., 2002). This model was based initially on observations in KtrA that charged NAD^+^ occluded the hinge groove of the dimer whereas NADH bound in a manner that facilitated hinge motion, thus reconciling ligand regulation directly with meaningful conformational changes in the protein. Now we show that the ancillary transporter-activating subunit KefF binds under the KTN hinge of KefC such that it promotes hinge opening and stabilizes this angle. We also suggest that KefC's KTN domains function as dimers without any propensity to form higher order assemblies, suggesting that such quaternary structures are not inherently necessary for activity.

One can ask whether the conclusions drawn with regard to KefC's KTN domain can be extrapolated to all KTN (RCK)-bearing regulatory complexes. Indeed their evolutionary relationship does not guarantee that their functional mechanism has necessarily also been conserved. However, comparing the structures of Kch channel KTN (RCK) domains, both open-hinged (MthK) and closed-hinged (*E. coli* Kch) conformations are represented (Figure 4A). With Ktr systems, we postulated previously as to what hypothetical hinge angle could support the formation of the tetrameric assemblies of KTN (RCK) domains that we had observed in solution through light scattering and analytical centrifugation studies (Roosild et al., 2002). By modeling dimer-dimer association via the critical protein interaction interfaces, we determined this angle to be nearly identical to the open hinge angle now seen with KefC (Figure 4A).

Our structure brings additional understanding as to still another mechanism by which these channels and transporters might be regulated. Kef systems have an additional level of regulatory complexity, being activated by GSH adducts and inhibited by reduced GSH. The structure of KefC-CTD reveals a novel arm structure that protrudes into the protein interaction interface, sterically prohibiting protein-protein interdomain associations as has previously been observed for these proteins. A binding pocket for GSH between this arm and the rest of the KTN domain suggests that the arm can either be stabilized in the obstructive conformation by a smaller GSH molecule or possibly destabilized by bulkier GSH adducts, modifying in turn the protein interaction interface's potential ability to associate with parts of the membrane domain, which might lead to activation of K^+^ efflux. This model is consistent with the observation that KefC activation is strongest with large bulky adducts, such as 5-membered rings and benzyl groups, and that small, hydrophilic adducts are less effective (Elmore et al., 1990). It also explains how the protein can respond to a wide variety of chemically diverse electrophiles through a single ligand binding site.

In other KTN-(RCK)-bearing permeases, as yet unidentified regulators might associate with the KTN (RCK) protein interaction interface, in a manner analogous to helices α7 and α8 within KefC, to shut down those systems. Although our knowledge of the complexity of KTN (RCK) domain-dependent K^+^ transporters and channels is incomplete, the two systems that are functionally best characterized (*E. coli* KefC and Trk) both involve additional subunits that have demonstrated regulatory roles. The Trk K^+^ uptake system has a fused KTN dimer in each TrkA subunit that associates with another essential protein, SapD (also known as TrkE; Harms et al., 2001). SapD proteins, based on their homology to ABC ATPases, are likely to also be dimeric (Chen et al., 2003). The system is nonfunctional if either TrkA or SapD is eliminated, and it is therefore conceivable that SapD plays a role equivalent to KefF with KefC.

This new structure of the KefC-KefF complex also assists in the debate regarding the higher oligomeric structures seen in other crystals of KTN (RCK) domains. Two alternative octameric ring hypotheses have arisen from structural studies of these domains. In MthK channels, RCK domains that are covalently linked to the pore-forming transmembrane domain are interlaced with separate RCK domains created from an internal start site (Met107) to regulate a single pore (Jiang et al., 2002). Modeled symmetrical rotation around the center axis of the flat protein interaction interface results in ring constriction as an elegant means of regulating channel flux, and the hinge in this scenario is considered “fixed.” Alternately, *B. subtilis* KtrA's KTN domain in multiple ring conformations that approximated the asymmetrical “squishing” of the ring along one axis (Albright et al., 2006) were found to bind two transmembrane transporter subunits. In these structures the hinge angle varies only slightly, with most of the conformational change attributed again to rotation of KTN dimers around the flat protein interaction interface. Although isolated KTN (RCK) domains likely have the ability to form such flexible octameric assemblies in vitro due to the inherent conformational mobility imparted by their architecture, the relevance of these structures to in vivo function remains unclear. Additionally, neither model explains by what means the directed rotation of the flat, hydrophobic protein interfaces might be effected to ultimately regulate ion conductance. Of the 15 known KTN (RCK) structures, only 7 have octameric ring organizations, and of those, only 1 has an octamer within the asymmetric unit (ASU), that one being the acknowledged artificial MthK structure (Table 2). Crystallographic symmetries (four-fold with one dimeric KTN (RCK) in the ASU; two-fold with two dimers in the ASU) need to be invoked to build the other observed octameric quaternary structures. What remains unknown is whether the apparent ready formation of octameric rings by solubilized KTN-(RCK)-bearing proteins might arise from the failure in recombinant overexpression systems to produce stoichiometric amounts of other structural, stabilizing components of these transporters and channels, equivalent to the role of KefF with KefC.

## Experimental Procedures

### Protein Expression and Purification

An N-terminally histidine-tagged construct of KefC-CTD (residues 401–620) linked to KefF (full length) with 12 intervening residues (TSGGLVPRGSSG) was built into a pET-28-derived vector. Production of KefC-CTD:KefF fusion protein followed standard laboratory protocols for recombinant bacterial protein expression and purification. In brief, freshly transformed BL21(DE3) *E. coli* colonies were cultured in Terrific Broth and induced with 0.1 mM isopropyl-β-D-thiogalactopyranoside at an O.D. of 1.0. Growth was continued overnight at 10°C–18°C. Cells were harvested and resuspended in 50 mM Tris (pH 8.0), 300 mM KCl, 10% glycerol, 10 mM imidazole with 1 mg/ml lysozyme. The bacteria were then disrupted by sonication on ice and membranes with other insoluble material were pelleted by high-speed centrifugation (100,000 × g). The complex was subsequently purified from the resulting supernatant using Ni-NTA affinity chromatography, applying an imidazole gradient (from 20 mM to 500 mM in a running buffer composed of 50mM Tris (pH 8.0), 300 mM KCl, and 10 mM β-mercaptoethanol) for elution to remove heterogeneous KefF fragments that possess a natural affinity to Ni^2+^. Purified protein was analyzed by SDS-PAGE, before and after overnight incubation with thrombin at 4°C to remove the polyhistidine affinity tag (the second thrombin motif within the linker of the fusion does not cleave under these conditions). Further purification was conducted using gel filtration chromatography over Superdex 200 resin. The final sample was verified to be homogenous by further SDS-PAGE experiments and dialyzed against a buffer as appropriate for either biochemical analysis or crystallization.

### Crystallization

Large-scale preparations of KefC-CTD:KefF fusion protein provided the starting material for initial crystallization trials of the cytoplasmic Kef regulatory domains. Purified KefC-CTD:KefF fusion protein was screened against the JCSG+ crystallization matrix (QIAGEN) with combinatorial supplementation of potential ligands NAD^+^/NADH and GSH/GSSG. An initial promising lead containing PEG 3350, MPD, and Mg^2+^ with NAD^+^ was optimized to produce three-dimensional crystals exceeding 50 microns in each dimension. The largest and best diffracting crystals were grown in 12% MPD, 10% PEG 3350, 40–100 mM MgCl_2_, 100 mM HEPES (pH 7.0), 1 mM NAD^+^ with 1 mM “HALESDIE” peptide (GenScript) added to the protein (5 mg/ml). Crystals frozen by submersion in liquid nitrogen after a few seconds incubation in cryoprotectant containing the above constituents, but with 25% MPD and also possessing 1 mM peptide displayed the lowest mosaicity, though it remains unclear whether the affects of adding the peptide are due to a specific interaction with KefC-CTD:KefF or nonspecific in nature, because no density for the peptide could be found in the electron density maps. In very similar conditions both orthogonal and trigonal crystals would grow.

### Data Collection and Processing and Structure Determination

Data were collected at Stanford Synchrotron Radiation Laboratory (SSRL) beamline 7-1 and showed potential for diffraction to 2.0 Å resolution. A complete, high-quality data set to 2.4 Å resolution was obtained, as was a lower-resolution data set on a smaller triangular crystal. Collected data were processed and reduced by the HKL2000 (Otwinowski and Minor, 1997) package with Denzo and Scalepack. The higher-resolution crystal was of the orthogonal space group P2_1_2_1_2_1_ with low mosaicity. The second crystal form belongs to trigonal space group P3_2_21. Molecular replacement phasing of the data obtained on the KefC-CTD:KefF fusion protein was attempted using Molrep (CCP4, 1994) with homology models of KefF, based on QR1 and QR2 (Li et al., 1995; Foster et al., 1999; Faig et al., 2000) and MdaB (Adams and Jia, 2006), and KefC-CTD, based on the KTN domains from *M. jannaschii* and *B. subtilis* (Roosild et al., 2002) created with Modeler (Sali and Blundell, 1993). Iterative cycles of model improvement with Refmac (CCP4, 1994), build-up of potential dimer orientations, and trial-and-error, eventually yielded phases sufficient to resolve density for the associated ligands and unmodeled residues. Modeling was performed using Coot (Emsley and Cowtan, 2004). KefC C-terminal residues 563–620 in one monomer and residues 578–620 in the other, the two C-terminal residues of KefF (‘HG’), and all expression tag and linker residues could not be built due to lack of electron density for these regions. Additionally, the nicotinamide ring and associated ribose sugar group of the NAD^+^ molecule bound to the KefC KTN domain could not be seen in the density maps and was left unmodeled. The final high-resolution structure was refined with Refmac to an R_factor_/R_free_ of 19.7%/23.9%, respectively, with approximately 91% of residues in most favorable regions of the Ramachandran plot as analyzed by Procheck (Laskowski et al., 1993). The model was further validated with Molprobity (Davis et al., 2007) scoring in the 91^st^ percentile. The trigonal structure was analyzed at medium resolution by molecular replacement and rigid body refinement of the high-resolution model. The atomic coordinates and structure factors of the high-resolution structure have been deposited in the Protein Data Bank (3EYW).

### Light-Scattering Studies

The KefC-CTD/KefF complex was analyzed using size exclusion chromatography on an Äcta Basic FPLC (GE) with an in-line MiniDAWN TREOS light scattering detector (Wyatt Technology) and Optilab rEX refractive index detector, in accordance with the Wyatt manual. Samples were run combinatorially with and without GSH, S-lactoyl-GSH, or N-ethylsuccinimido-S-glutathione, and with or with NAD^+^ or NADH. In all combinations the Kef regulatory complex was found to be dimeric. All samples were run at a pH of 7.5 because the KefC-CTD:KefF fusion complex rapidly precipitates completely out of solution as pH drops below 7.0. The running buffer for these experiments was composed of 50 mM HEPES (pH 7.5), 150 mM KCl, 1 mM of either DTT or TCEP, and 1 mM of any supplemented ligands.

### KefC Efflux Experiments

Site-directed mutants of Kef system expression plasmids (pSM12 and pKefFKefCHis_6_; Miller et al., 1997) were generated using the QuikChange protocol (Stratagene) and transformed into MJF335 (a GSH-deficient strain of *E. coli*, allowing for control of the concentration of this ligand through the growth medium). Functional analysis of KefC K^+^ efflux was conducted as previously described (Miller et al., 1997). In brief, cells were grown in 120 mM K^+^ medium in the presence or absence of GSH (1 mM). Samples were incubated in stirred glass vessels at 37°C and divided equally. Control samples were observed for spontaneous K^+^ loss, whereas 0.5 mM NEM was added to the remaining culture to activate the transporter. Samples were removed at various time intervals and the retained cellular K^+^ content was measured by flame photometry (Corning 400) as previously detailed (Elmore et al., 1990).

### Blue Native Gel Electrophoresis

Bacterial membranes with overexpressed full-length KefC corresponding to 60 μg protein were solubilized with 1.5% dodecylmaltoside in a total volume of 30 μl for 30 min on ice. After 10 min centrifugation, 17.7 μl supernatant was mixed with 6.2 μl 4x native sample buffer (Invitrogen) with 1.1 μl 5% Coomassie G250. This mixture was applied to a preformed native Novex 4%–16% BisTris gel (Invitrogen). The electrophoresis, blotting, and detection were performed as described previously (Rasmussen et al., 2007).

### KefC Suppressor Analysis

Second site suppressors of KefC KTN mutants V427A and R416S were isolated as described previously (Miller et al., 2000) as colonies able to grow on media containing < 0.1 mM KCl. A single clone of each such colony was tested for the ability to demonstrate NEM-elicited efflux to verify the retention of KefC activity. Identified suppressors included several within the KTN domain (F407L, E520G, A522V, and G526V), as well as several insertion sequence mutations in either the promoter region 5′ of *kefF* or within the gene itself, in addition to the KefF destabilizing point mutations Q62L and L95R.

## Figures and Tables

**Table 1:** Summary of Crystallographic Data and Model Refinement Statistics

Diffraction Data		

Crystal form	A	B
Source	SSRL 7-1	SSRL 7-1
λ	1.00 Å	1.00 Å
Space group	P2_1_2_1_2_1_	P3_2_21
Cell constants	a = 64.58 Å	a = 86.11 Å
	b = 85.17 Å	b = 86.11 Å
	c = 189.03 Å	c = 241.16 Å
Mosaicity	0.33°	0.69°
Resolution	50-2.40 Å (2.49-2.40 Å)	50-4.00 Å (4.14-4.00 Å)
R_merge_	9.1% (39.1%)	8.0% (25.3%)
I/σ	18.4 (5.7)	12.1 (2.9)
Completeness	99.7% (100.0%)	97.4% (86.0%)

Model Refinement		

Number of reflections	39,230	9,344
Number of monomers/A.U.	2	2
Atoms/A.U.	5827	4 rigid bodies
Protein	5493	
Ligand	153	
Water	181	
R_cryst_	19.7%	33.9%
R_free_	23.9%	33.2%
Rmsd bond lengths	0.013 Å	
Rmsd bond angles	1.41°	
Ramachandran statistics		
Most favored regions	91.2%	
Additionally allowed regions	8.8%	

**Table 2:** Summary of Known KTN (RCK) Structures

Protein Family	Organism	Form	Protein Data Bank ID	Space Group	Asymmetric Unit	Extended Oligomeric Assembly	Illustration
Kef	*E. coli*	“Dimer”	3eyw	P2_1_2_1_2_1_	1 dimer	Dimer	
		“Dimer of dimers”		P3_2_21	1 dimer	Dimer	
Kef	*V. parahaemolyticus*		3c85	P2_1_2_1_2_1_	2 dimers	Dimer	
KtrA	*B. subtilis*	“Square”	1lsu, 2hmw	I422	1 dimer	Octamer	
		“Diamond”	2hmt, 2hmu, 2hmv	P422	1 dimer	Octamer	
		“Rectangle”	2hms	P3_1_12	2 dimers	Octamer	
KtrA	*M. jannaschii*	A	1lss	P2_1_2_1_2	2 dimers	Octamer	
		B		I4_1_22	1 dimer	Octamer	
		C		P2_1_2_1_2_1_	1 dimer	Zigzag	
KtrA	*T. maritima*		2g 1u	P2	monomer	Zigzag	
Kch	*E. coli*		1id1	P4_1_	1 dimer	Coil	
Kch	*M. thermoautotrophicum*	TM form	1lnq	P6_1_	octamer	Octamer	
		“Octameric ring”	2fy8	C222_1_	4 dimers	Octamer	
		“Hexameric ring”	2aem, 2ogu	H32	monomer	Hexamer	
		“Calcium bound”	2aef, 2aej	P2_1_	1 dimer	Dimer	

Fifteen unique structures are now known from three different protein families (Ktr, Kch, Kef) with two or three different representatives of each. Less than half of these structures have an octameric ring assembly, and only one (the full-length, tail-to-tail interlaced artifactual MthK structure; 1lnq) forms a ring without invoking crystallographic symmetries. In contrast, the dimeric hinged assembly is inviolate. For calculating “extended oligomeric assembly,” only contacts through the KTN (RCK) protein interaction interface were considered. In the illustration, ˆ represents a single canonical dimer, and each orange semicircle stands for a protein interaction interface.

## Accession Numbers

Coordinates have been deposited in the RCSB Protein Data Bank with the accession code 3EYW.
